# Hydrogen Peroxide in the Pulp Chamber and Color Change in Maxillary Anterior Teeth After In-Office Bleaching

**DOI:** 10.1590/0103-6440202405793

**Published:** 2024-10-28

**Authors:** Alexandra Mena-Serrano, Sandra Sanchez, María G. Granda-Albuja, Michael Willian Favoreto, Taynara de Souza Carneiro, Deisy Cristina Ferreira Cordeiro, Alessandro D. Loguercio, Alessandra Reis

**Affiliations:** 1 School of Dentistry, Universidad de Las Américas, Quito, 170125, Ecuador; 2 Laboratorios de Investigación. Universidad de Las Américas, Quito, 170125, Ecuador; 3 Department of Restorative Dentistry, School of Dentistry, State University of Ponta Grossa, Brazil; 4 Area of Stomatology, IDIBO research group, Health Sciences Faculty, Rey Juan Carlos University, Alcorcón, Madrid, Spain

**Keywords:** Tooth bleaching, dental enamel permeability, color shade, tooth permeability, hydrogen peroxide

## Abstract

While the literature has noted variations in hydrogen peroxide (HP) permeability, and consequently, tooth sensitivity among different types of anterior teeth, there is a scarcity of research on this specific topic. This study evaluated HP permeability and color change (CC) in different groups of human maxillary anterior teeth (canines, lateral incisors, and central incisors) after in-office bleaching with HP at 35%. Thirty teeth maxillary anterior (n=10) were bleached with HP at 35% gel in one session of three 15-minutes applications. Buccal thickness (mm) was measured from images obtained using cone beam computed tomography. The concentration of HP (µg/mL) that reached the pulp chamber was assessed using UV-Vis spectrophotometry. CC (WID, ∆Eab, and ∆E00) was evaluated before and after bleaching with a digital spectrophotometer. One-way ANOVA and Tukey’s test were applied to statistically evaluate the data for buccal thickness, HP permeability, and CC (α=0.05). Comparison between thickness and HP permeability was performed using Pearson's correlation. Thicker teeth, such as canines, had lower HP amounts inside the pulp chamber compared to central and lateral incisors. Despite the significant effect of buccal thickness on HP permeability (p<0.05), no correlation was found between the two factors. CC was similar across tooth types (p>0.05). The difference in buccal thickness among the superior anterior teeth does not interfere with CC. However, a thinner buccal wall thickness is associated with greater HP permeability detected in the pulp after in-office bleaching.

## Resumo

Embora a literatura tenha observado variações na permeabilidade do peróxido de hidrogênio (PH) e, consequentemente, na sensibilidade dental entre diferentes tipos de dentes anteriores, há escassez de pesquisas sobre esse tema específico. Este estudo teve como objetivo avaliar a permeabilidade do PH e a mudança da cor (MC) em diferentes grupos de dentes anteriores superiores humanos (caninos, incisivos laterais e incisivos centrais) após clareamento de consultório com PH a 35%. Trinta dentes anteriores superiores (n=10) foram clareados com gel de PH a 35% numa sessão de três aplicações de 15 minutos. A espessura vestibular (mm) foi medida em imagens de tomografia computadorizada de feixe cônico. A concentração de HP (µg/mL) na câmara pulpar foi avaliada por espectrofotometria UV-Vis. A MC (WI_D_, ∆E_ab_ e ∆E_00_) foi avaliada antes e após o clareamento com espectrofotômetro digital. Aplicou-se ANOVA unidirecional e teste de Tukey para avaliar estatisticamente os dados de espessura bucal, permeabilidade PH e MC (α=0,05). A comparação entre espessura versus permeabilidade PH foi realizada com correlação de Pearson. Dentes mais grossos, como os caninos, apresentaram menor quantidade de HP no interior da câmara pulpar do que os incisivos centrais e laterais. Apesar da espessura vestibular afetar significativamente a permeabilidade do PH (p<0,05), não foram encontradas correlações entre ambos os fatores. A MC foi semelhante, independente do tipo de dente (p>0,05). A diferença na espessura vestibular dos dentes anteriores superiores não interfere na MC. Porém, quanto mais fina a espessura da parede vestibular, maior será a permeabilidade do PH detectada na polpa após o clareamento de consultório.

## Introduction

A smile featuring well-aligned maxillary anterior teeth, with the correct color, position, and shape, is considered important for patients ^1^. Professionals should take these characteristics into consideration by professionals, especially given the influence of patient demand influenced by social media ^2^. Dental bleaching is highly recommended when color changes are desired ^1^. Among several techniques available, in-office dental bleaching is often the most appropriate choice, as it provides faster results ^3^.

In this technique, the patient undergoes the procedure inside the dental office. After protecting soft and gingival tissues, the professional applies a high concentration (30-40%) of hydrogen peroxide (HP) over the surface of the teeth for 30-50 minutes in each session ^3^. Generally, due to the lower molecular weight of HP, it can diffuse easily through the enamel and dentin ^4^. This chemical agent acts on organic structures, primarily in dentin, promoting breakdown and, consequently, the whitening effect ^4^. However, HP is not confined solely to hard tissues; it diffuses through enamel and dentin until it reaches the pulp chamber in smaller but sufficient amounts to produce tooth sensitivity ^4^ ^,^ ^5^. This very common and temporary side effect after bleaching can be explained by inflammatory processes, partial local necrosis of pulp cells, and moderate dentin formation (5-8) due to oxidative stress.

Various factors can influence the penetration of HP, including different concentrations, pH, viscosity, and composition of bleaching agents ^9^ ^,^ ^10^ ^,^ ^11^. Another factor, less explored in the literature, is variation in tooth size. It is expected that teeth of smaller sizes, indicating lower buccal thickness, will exhibit higher penetration of HP into the pulp chamber compared to larger size teeth with higher buccal thickness. Consequently, this may lead to a greater inflammatory process within the pulp chamber in the former ^6^. However, there is no consensus regarding tooth sensitivity when teeth of different sizes are evaluated. While some clinical studies have reported more intense intensity of tooth sensitivity in smaller size teeth ^12^ ^,^ ^13^, others have not shown any significant difference, regardless of the size of the teeth ^14^.

However, some studies evaluating bleaching effects in vitro utilize sections of bovine teeth with simulated pulp chambers in varying thicknesses. While these studies have provided valuable insights, the use of human teeth with their natural pulp chambers can offer a closer approximation to clinical scenarios ^5^ ^,^ ^15^ ^,^ ^16^ ^,^ ^17^ ^,^ ^18^ ^,^ ^19^ ^,^ ^20^ ^,^ ^21^ ^,^ ^22^ ^,^ ^23^. On the other hand, premolars are commonly chosen for experiments involving human tooth crowns, primarily due to their extraction during orthodontic procedures ^9^ ^,^ ^11^ ^,^ ^24^ ^,^ ^25^ ^,^ ^26^ ^,^ ^27^ ^,^ ^8^. However, a closer examination of the literature reveals that tooth sensitivity is predominantly reported in anterior teeth, rather than premolars ^12^ ^,^ ^13^. Therefore, it seems appropriate to investigate whether the amount of HP within the pulp chamber varies when different superior anterior teeth (canines, lateral incisors, and central incisors) undergo in-office bleaching. This study represents the first attempt to correlate tooth thickness with HP permeability into the dental pulp.

Therefore, the aim of the present study was to evaluate the amount of HP inside the pulp chamber and color change in groups of extracted human maxillary anterior teeth, specifically canines, lateral incisors, and central incisors, after undergoing in-office bleaching with 35% HP. We tested the following primary research hypothesis: [1] there will be a difference in the amount of HP inside the pulp chamber when different teeth are subjected to in-office bleaching. Additionally, we tested the following secondary hypothesis: [2] there will be a difference in color change after in-office bleaching among different teeth evaluated.

## Material and Methods

### Ethics committee approval and selection of teeth and inclusion and exclusion criteria

This study was submitted to the local ethics committee, which approved it under agreement number (5.740.189). This study used thirty human maxillary anterior (10 canines, 10 lateral incisors, and 10 central incisors) obtained from Human Teeth Local Bank. The teeth were observed under a microscope at 10 x magnification (Eclipse E200, Nikon, Tokio, Japan). The selected teeth were required to have a baseline Whiteness Index for Dentistry (WI_D_) ^30^ 25 units or smaller. The WI_D_ was measured by taking the color parameters obtained with the digital spectrophotometer Vita EasyShade (Vita Zahnfabrik, Bad Säckingen, Germany). Exclusion criteria included teeth with endodontic treatment, incomplete root formation, presence of previous restorations, caries, and severe tooth discoloration (such as tetracycline stains or fluorosis).

### Sample size calculation

An earlier study ^11^ showed that the amount of HP detected in the pulp chamber when premolar specimens were subjected to a one-session of in-office bleaching protocol with 35% HP gel was on average 1.16 ± 0.34 µg/mL. Using a two-tailed test with 5% of alpha and 90% power, to detect a 50% difference between groups, sample sizes of at least eight teeth should be tested in each group. To prevent possible loss of teeth during bleaching procedures, two extra teeth were added per group.

### Specimen preparation

The specimens were prepared according to the previous study in the literature, and it was briefly described in this section ^9^ ^,^ ^11^ ^,^ ^26^ ^,^ ^27^ ^,^ ^28^. The roots of the teeth were cut approximately three millimeters from the enamel-cement junction. After removing the pulp tissue, the entrance of the pulp chamber (radicular area) was carefully expanded using a spherical drill, without touching the inner vestibular region of the pulp chamber. Following the procedure, the region was thoroughly rinsed with distilled water. It is important to note that this process does not modify the internal region of the pulp chamber and pulp horns. This was done with the purpose to introduce inside the pulp chamber around 25 μL of solution using a micropipette.

### Thickness of specimens

Transverse images from cone beam computed tomography (CBCT) were acquired perpendicular to the longitudinal axes of the teeth, while sagittal and coronal images were obtained parallel to the longitudinal axes of the teeth after software processing. These images were acquired using a CBCT scanner (Phillips Brilliance 64, Philips Medical Systems, Eindhoven, Nederland), with a voxel size of 125 μm, operated at 120 kV and 350mAs by an experienced radiologist, following the manufacturer's recommended protocol to ensure high-quality images, using an exposure time of 2.5 seconds. The acquired data were then converted to the DICOM (Digital Imaging and Communications in Medicine) format for the subsequent measurement of buccal tooth thickness using the Philips Brilliance™ CT software (Philips Medical Systems, Eindhoven, Nederland), measuring the distance from the pulpal horn to the outermost buccal surface ^31^. Since previous histopathological studies have shown the presence of tissue damage in the horn area in human teeth treated with in-office bleaching ^6^ ^,^ ^7^ ^,^ ^8^, the mentioned distance was selected to represent the path for penetration of HP into the pulp.

### Obtaining the analytical curve

First, a standard analytical curve was obtained from a 5.000 μg/mL stock solution prepared from a concentrated solution (37% HP, Thermo Fisher Scientific, Madrid, Madrid, Spain). This solution was diluted in an acetate buffer solution (pH = 4) and titrated using traditional methods with a potassium permanganate solution to determine the analytical grade and the actual concentration of the solution ^11^. From this concentration, serial volumetric dilutions of 0.000-0.397 μg/mL were performed to draw the analytical curve. A UV-Vis spectrophotometer (UV-1280, Shimadzu, Japan) was used to know the concentrations of HP and finally obtain a standard reference line for the extrapolation of the study samples’ results (R = 0.994; not shown data).

### Treatment bleaching protocols

A single calibrated and experienced operator was responsible for the application of all treatment bleaching protocols. Teeth were fixed vertically to the silicone base (Speedex, Coltène/Whaledent AG, Feldwiesenstrasse, Altstätten, Switzerland). The contour of the buccal surface was delimited with a light-cured resin dam, enclosing an area of 6 mm x 6 mm (Top dam; FGM Dental Products, Joinville, SC, Brazil) ^(^ ^9^ ^,^ ^11^ ^,^ ^26^ ^,^ ^27^ ^,^ ^28^. The 35% HP bleaching gel was used as an in-office product (Whiteness HP, FGM Dental Products, Joinville, SC, Brazil) in a single session, and it was applied to the enamel buccal surface three times at 15-minute intervals, according to the manufacturer’s recommendation. The application of the whitening gel was sufficient to cover the area to be performed the bleaching procedure, within the delimitation created with the gingival barrier. Consequently, comparable amounts of gel were used in all specimens, irrespective of tooth type.

### Hydrogen peroxide permeability in the pulp chamber

As in previous studies (9,11,26-28), after the bleaching procedure, the acetate buffer solution in the pulp chamber of each sample was removed and transferred to a glass tube immediately after the session. To ensure the complete removal of HP, the pulp chamber was rinsed four times with 25 μL of acetate buffer. This rinse solution was transferred to the same glass tuve. Following this, 100 μL of 0.5 mg/mL (Leucocrystal Violet, Sigma Chemical Co., St Louis, MO, USA), 50 μL of 1 mg/mL of horseradish peroxidase (Peroxidase Type VI-A, Sigma Chemical Co., St. Louis, MO, USA) and deionized water (2.725 μL) were added to the glass tube. This sequence was repeated separately for each tooth at different times. The resulting solution was measured using a UV-Vis spectrophotometer (UV-1280, Shimadzu). According to the Beer-Lambert Law, absorbance is directly related to the concentration of the solute and the optical path length of the light beam through the solution ^29^. Therefore, the concentration of HP (μg/mL) was determined by comparing the absorbance with the previously obtained calibration curve.

### Color change evaluation

The color of all groups was measured before starting any procedure and one week after ^26^, using a digital spectrophotometer (VITA Easyshade Advance 5.0, VITA Zahnfabrik, Bad Säckingen, Baden-Württemberg, Germany). As described previously ^28^, to standardize the position of the spectrophotometer for the different measurements, guides were made using dense condensation silicone (Speedex, light green color, Coltène/Whaledent AG), with a 6 mm diameter window created in the middle one-third of the buccal surface for each specimen using a metal device. Color measurements were performed in triplicate, and the average of each measurement was used for statistical purposes.

The color parameters (L*, a*, and b*) were recorded through the tip of the device inserted into the silicone guide. The color change before (baseline) and after bleaching was determined by the difference between the colors measured with the spectrophotometer, using the WI_D_ ^30^, ∆E_ab_ ^32^, and ∆E_00_ ^33^. Perceptual changes ^34^ were considered significant if the differences between the initial and post-bleaching measurements were WI_D_ > 2.6 ^35^, ∆E_ab_ > 2.7 and ∆E_00_ > 1.8 ^36^. Throughout all the experiment, the specimens were immersed in saliva, as previously described ^(^ ^9^ ^,^ ^28^.

### Statistical analysis

Firstly, the data were tested for normality using the Shapiro-Wilk test and for equality of variances using Bartlett’s test (data not shown). Subsequently, the data on buccal thickness (mm), HP concentration detected in the pulp chamber (µg/mL), and color change (baseline WID, WID, ∆Eab, and ∆E00) were statistically analyzed using one-way ANOVA followed by the Tukey test (α = 0.05). Direct comparisons between buccal thickness and HP concentration in the pulp chamber were performed using Pearson’s correlation (α = 0.05).

## Results

The buccal thickness was significantly different among the maxillary anterior teeth evaluated, with significant differences observed only when comparing canines, the thicker teeth, with other teeth (p = 0.04; Table 1). HP was detected in the pulp chamber of all types of teeth. However, the concentration was significantly lower for the canine group (p = 0.02; Table 1). Pearson's correlation indicated a weak negative correlation (r = -0.49), suggesting that as one variable increased, the other decreased by a similar magnitude, though this was not statistically significant (p = 0.99).

**Table 1:** Means and standard deviations of the buccal tooth thickness (mm) and HP concentration (µg/mL) of the different tooth types, as well as statistical analyses (*)

	Thickness (mm)	HP inside pulp chamber (µg/mL)
Central incisors	2.3 ± 0.2 b	0.403 ± 0.093 A
Lateral incisors	2.1 ± 0.3 b	0.471 ± 0.059 A
Canines	2.9 ± 0.2 a	0.318 ± 0.083 B

*The same letters indicate similar statistical differences between averages (ANOVA p < 0.05).

**Figure 1:**
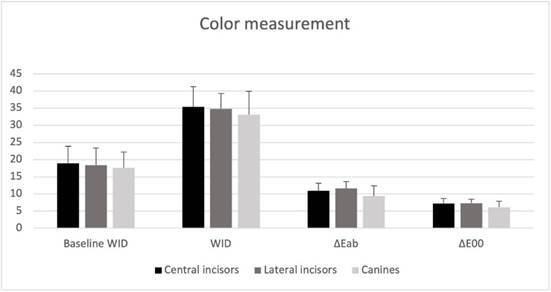
Color measurement of different tooth types. WI_D_ = Whiteness Index For Dentistry ^30^; ΔE_ab_,= CIElab1976 ^32^ and ΔE_00_. = CIEDE2000 ^33^

Table 2 and Figure 1 show that the baseline WI_D_ tooth color was similar across all groups (*p* > 0.05). After bleaching (Table 2 and Figure 1), all parameters evaluated for color change showed significant bleaching effects (*p* < 0.05). However, when comparing all groups, no significant differences were observed in the bleaching outcomes for any of the color parameters evaluated (p > 0.36; Table 2 and Figure 1).

**Table 2:** Means and standard deviations of the color change in baseline WI_D_, WI_D,_ ΔE_ab_, and ΔE_00_ of the different tooth types, as well as statistical analyses (*)

	Baseline WI_D_	WI_D_	ΔE_ab_	ΔE_00_
Central incisors	18.9 ± 5.0 a	35.4 ± 5.9 A	10.9 ± 2.2 ^a^	7.2 ± 1.5 ^A^
Lateral incisors	18.4 ± 5.0 a	34.8 ± 4.4 A	11.6 ± 2.0 ^a^	7.3 ± 1.1 ^A^
Canines	17.6 ± 4.6 a	33.1 ± 6.8 A	9.4 ± 2.9 ^a^	6.1 ± 1.7 ^A^

*The same lower case, capital, superscript letters indicate similar statistical differences between groups (ANOVA p < 0.05).

## Discussion

The study results support the primary hypothesis, as there was a difference in the HP levels inside the pulp chambers. However, the secondary hypothesis was rejected, as no significant difference in color was detected among the groups.

Upper anterior teeth are often subject to aesthetic demands, and bleaching is essential for improving color and appearance. However, HP diffusion into the pulp chamber is an undesirable but inevitable event that occurs during and after bleaching procedures ^4^. Several studies have provided valuable information on how application modes ^23^ ^,^ ^24^ ^,^ ^26^, composition ^11^ ^,^ ^25^, and the physico-chemical characteristics of the bleaching gel ^9^ ^,^ ^28^ can influence the amount of HP detected in the pulp chamber.

Individual characteristics of the teeth are also important to consider for bleaching procedures. Buccal thickness affects the amount of HP that enters the pulp chamber. Central and lateral incisors have thinner enamel-dentine buccal surfaces than canines, and the former showed more HP inside the pulp chamber than canines after in-office bleaching.

It can be argued that in thicker teeth, such as canines, a greater area of organic substrate is available for oxidation. The free radicals react more with the organic structure present in thicker substrates, resulting in a lesser amount of HP inside the pulp chamber. Conversely, teeth with thinner enamel are more likely to experience inflammatory processes in the pulp tissue due to the higher amount of HP that can reach it. This can cause oxidative stress in the tissue, leading to alterations in its morphology and a decrease in cell viability and regeneration. Consequently, this can result in varying degrees of tooth sensitivity, depending on the patient ^5^ ^,^ ^22^.

The relationship between the buccal thickness of the dental substrate and the amount of HP detected in the pulp chamber after in-office bleaching was also observed in previous studies ^5^. The authors simulated various enamel/dentin thicknesses by polishing bovine teeth to create 4-mm and 2.3-mm enamel/dentin discs representing maxillary first premolars and mandibular central incisors, respectively ^5^. In contrast, we utilized a more realistic scenario by using anterior human extracted teeth without any preparation. The results demonstrated that even small differences in thickness (0.8 mm and 0.6 mm between canines and lateral incisors and central incisors, respectively) could lead to significant differences in the amount of HP detected in the pulp chamber.

Despite similar results being observed compared to the previous study ^5^, the amount of HP reaching the pulp chamber is markedly different in the present study. While the aforementioned study ^5^ reported around 7 µg/mL for thicknesses similar to lower incisors and 5 µg/mL for thicknesses similar to premolars, our study found significantly lower concentrations. Several methodological differences, including the type of teeth used and the format of specimens, are likely responsible for the observed differences.

When trimming the teeth to achieve the desired thicknesses, the authors compensated for the smaller thickness of bovine enamel in the premolar group by leaving a thicker dentin substrate compared to human premolars ^5^. The thickness of dentin might allow more passage of HP than a more mineralized, less permeable substrate such as enamel. Additionally, the previous study involved the fabrication of an artificial pulp chamber where 1 mm of the medium solution was in contact with the enamel-dentin disk ^5^. In our study, a smaller amount of buffer solution could be used due to the natural pulp chamber's constraints, necessitating subsequent rinsing to complete the total solution volume for analysis.

This is the first study to attempt to correlate tooth thickness with HP permeability into the dental pulp. Although no correlation was found between the thickness of various teeth and their permeability, it appears that thickness may not be a key factor in explaining the amount of HP reaching the dental pulp. Despite controversial results regarding tooth sensitivity and size ^12^ ^,^ ^13^ ^,^ ^14^, only one clinical study has correlated the measured tooth sensitivity of maxillary central incisors with their thickness, finding no significant correlation ^14^. This lack of correlation may be attributed to various factors, including specific characteristics such as the patient's age. Previous studies have shown that younger teeth typically exhibit thinner dentin and wider dentinal tubules compared to older teeth. Additionally, younger teeth generally have less secondary dentin than older teeth ^8^.

One characteristic of the reduction in pulp chamber area is the continuous deposition of a dentin matrix rich in physiological collagen (secondary dentin) by odontoblasts ^8^, which occurs with age or as a response to occlusal trauma. In our study, the statistical differences observed between central and lateral incisors compared to canines suggest that the path to the pulp chamber was longer in the latter, likely due to ongoing dentin deposition. Furthermore, apart from anatomical differences, canines undergo a longer maturation process and are more subjected to occlusal trauma, as previously mentioned. Consequently, canines tend to appear darker due to the increased amount of dentin present. This increase in thickness, along with decreased permeability from peritubular dentin deposition in older teeth or due to trauma, is expected to offer additional protection to the pulp ^8^.

Regarding color change, the present study used the WI_D_ (*Whiteness Index for Dentistry*) to assess baseline tooth color and color change efficacy, following more recent recommendations ^30^ ^,^ ^35^. The use of baseline WI_D_ and ΔWI_D_ are newer tools ^30^ ^,^ ^35^ recommended for measuring tooth bleaching effectiveness based on the CIELab color space. This updated formula reduces the probability of error in assessing whiteness ^30^ ^,^ ^35^ and enables significant observation of color improvement for all teeth after just one session. Traditionally, previous studies have evaluated color change using only ΔE_ab_ ^5^ ^,^ ^20^ ^,^ ^21^.

The ΔE_ab_ metric permits comparison with data from earlier studies ^5^ ^,^ ^20^ ^,^ ^21^; however, the ΔE_00_ can indicate color differences perceived by the human eye more accurately than ΔE_ab_ ^36^. The ΔE_00_ considers not only chroma, hue, and lightness, weighting functions; it proposes potential interactions between hue differences and chroma to enhance performance for blue and gray colors ^33^. While both ΔE_ab_ and ΔE_00_ metrics are used in dentistry, they assume equal influence for all color coordinates. Consequently, these values do not indicate whether the color change of dental structures moved towards lighter or darker shades. This is why the ΔWI_D_, which measures the level of whiteness, is directly relevant in bleaching studies. Canines, being thicker teeth, are expected to be darker and therefore more challenging to whiten due to their inherent characteristics. However, our findings revealed no statistical difference in tooth color at baseline or after bleaching.

A recent study highlighted that the color of the silicone guide itself can influence measurement outcomes. The authors ^37^ suggested (although not evaluated in their design) using transparent guides for future research in the bleaching field to minimize interference in the results. It is important to note that, due to our unawareness of this potential interference, we used light green silicone. Any potential impact of the colored silicone would have been consistent across both groups, not affecting the study’s internal validity.

Several limitations should be considered. While the study focused on relevant teeth for bleaching procedures, not all tooth types were included in the analysis. Additionally, due to the challenge of obtaining anterior sound teeth, primarily teeth from older patients were used, which may limit the generalizability of the findings. The age of the patients whose teeth were obtained can also be considered a limitation, as only older teeth were included in the study. Despite efforts made during the application of the bleaching gel, the exact quantity of gel was not measured beforehand, which could be considered a limitation of the present study.

In summary, human teeth with smaller buccal thickness and thinner enamel exhibit greater penetration of hydrogen peroxide into the pulp chamber following in-office bleaching. These findings suggest that, in clinical practice, such teeth may be more prone to developing adverse effects such as tooth sensitivity.

## Conclusions

The bleaching pattern observed with in-office bleaching appears to be consistent across all tooth sizes. However, it is noteworthy that only maxillary canines, which are thicker, showed a lower amount of hydrogen peroxide (HP) detected in the pulp chamber compared to maxillary central and lateral incisors.
